# Phytoextraction efficiency of *Pteris vittata* grown on a naturally As-rich soil and characterization of As-resistant rhizosphere bacteria

**DOI:** 10.1038/s41598-021-86076-7

**Published:** 2021-03-24

**Authors:** M. L. Antenozio, G. Giannelli, R. Marabottini, P. Brunetti, E. Allevato, D. Marzi, G. Capobianco, G. Bonifazi, S. Serranti, G. Visioli, S. R. Stazi, M. Cardarelli

**Affiliations:** 1 IBPM-CNR, Dip. Biologia e Biotecnologie, Sapienza Università di Roma, P.le A. Moro 5, 00185 Rome, Italy; 2 Dip. Biologia e Biotecnologie, Sapienza Università di Roma, 00185 Rome, Italy; 3 Department of Chemistry, Life Sciences and Environmental Sustainability, University of Parma, Parco Area delle Scienze 11/A, 43124 Parma, Italy; 4 Department for Innovation in Biological, Agri-Food and Forestry Systems (DIBAF), University of Viterbo, Via San Camillo de Lellis snc, 01100 Viterbo, Italy; 5 Dip. Ingegneria Chimica Materiali Ambiente, Sapienza Università di Roma, 00184 Rome, Italy; 6 Department of Chemical, Pharmaceutical and Agricultural Science (DOCPAS), University of Ferrara, 44121 Ferrara, Italy

**Keywords:** Biotechnology, Microbiology, Molecular biology, Plant sciences, Environmental sciences, Optics and photonics

## Abstract

This study evaluated the phytoextraction capacity of the fern *Pteris vittata* grown on a natural arsenic-rich soil of volcanic-origin from the Viterbo area in central Italy. This calcareous soil is characterized by an average arsenic concentration of 750 mg kg^−1^, of which 28% is bioavailable. By means of micro-energy dispersive X-ray fluorescence spectrometry (μ-XRF) we detected As in *P. vittata* fronds after just 10 days of growth, while a high As concentrations in fronds (5,000 mg kg^−1^), determined by Inductively coupled plasma-optical emission spectrometry (ICP-OES), was reached after 5.5 months. Sixteen arsenate-tolerant bacterial strains were isolated from the *P. vittata* rhizosphere, a majority of which belong to the *Bacillus* genus, and of this majority only two have been previously associated with As*.* Six bacterial isolates were highly As-resistant (> 100 mM) two of which, homologous to *Paenarthrobacter ureafaciens* and *Beijerinckia fluminensis,* produced a high amount of IAA and siderophores and have never been isolated from *P. vittata* roots. Furthermore, five isolates contained the arsenate reductase gene (*ars*C). We conclude that *P. vittata* can efficiently phytoextract As when grown on this natural As-rich soil and a consortium of bacteria, largely different from that usually found in As-polluted soils, has been found in *P. vittata* rhizosphere.

## Introduction

Arsenic (As) has received considerable attention in recent years because of As-contamination of water and soils throughout the world and its high toxicity to humans, animals and plants ^1^. Arsenic in the environment can be due to natural sources such as the weathering of rocks and volcanic material, but its presence is increasing due to anthropic activities such as the use of pesticides, industrial waste and smelting. A soil is defined polluted if the contamination is caused by man, or naturally contaminated if the contamination is due to geogenic sources, but in both cases As can pose risks for human health and the environment.

Arsenic is present in the environment both in organic and inorganic forms and in various oxidation states.

The inorganic trivalent and pentavalent forms of As are the most common in water and are also more toxic than the organic forms ^2,3^.

The stability of As compounds in the soil and their mobility in the water–soil–plant system are influenced by the physico-chemical and biochemical properties of the soil, such as texture, pH, redox potential, presence of exchangeable ions acting as competitors, organic matter content and biological activity ^2,4–6^.

In the soil, the quantity of bioavailable As is lower than the total content since the non-metal adsorbed on the soil colloids forms, over time, increasingly stable surface complexes ^4^. The bioavailability of the non-metal depends greatly on the soil–water–plant system ^2^.

The study of areas characterized by hydrothermal phenomena is particularly important because of the excessive exploitation of deep aquifers which lead to the mobilization of various elements including As. This is the case of some areas in central Italy, where the volcanic and hydrothermal origin leads to high concentrations of As in the soil ^7^.

Phytoextraction—the removal of elemental contaminants by plants—is an efficient environmentally-friendly technology for cleaning up As contaminated soils, and is an effective strategy for tackling the continuous release of As which occurs in As-rich natural soils ^8^. Phytoextraction relies on the ability of hyperaccumulator plants to extract As from the soil and sequester it in the aerial parts of the plant. The fern *Pteris vittata* is particularly effective for the remediation of soils contaminated by As because it is capable of transporting As, concentrations up to 100 times those present in the soil, to the fronds. ICP-OES analysis, which requires ion extraction from *P. vittata* plants, is currently being used to determine the accumulation of As in fronds after several months of growth on the contaminated soil. Recently, the non-destructive μ-XRF technology, which does not necessitate ion extraction, has been successfully to analyze the distribution of As, and other inorganic elements, in different organs of the plant ^9–11^. This technology has been used, alone or in combination with ICP-OES or scanning electron microscopy and revealed that the As levels in soils impacted As accumulation in *P. vittata* fronds. Indeed, high As levels enhanced root-to-frond As translocation ^12^.

The efficiency of As removal by *P. vittata* depends not only on the characteristics of the soil but also on bacteria associated with the plant rhizosphere ^13,14^. Bacteria can enhance *P. vittata* phytoextraction by providing specific compounds, such as the phytohormone indole acetic acid (IAA) which promotes plant growth ^15^, or siderophores, which generally increase iron uptake. Siderophores can also solubilize As adsorbed on Fe-oxides thus releasing As for the plant ^16,17^. In addition, a further increase in plant As accumulation is caused by As-resistant bacteria which are able to reduce AsV to AsIII, that is then extruded by bacterial cells utilizing an AsIII efflux pump. Bacteria utilize an AsV-reductase, coded by *arsC* which belongs to the *ars* operon, in this process ^18^.

*P. vittata* is able to produce more root exudates than those produced by the non-hyperaccumulator fern *Nephrolepis exaltata*. These root exudates, which consist mainly of phytic and oxalic acids, can select and sustain bacterial growth as well as help *P. vittata* mobilize As ^19^. Indeed Yang et al. ^20^ showed that the isolated *Pseudomonas vancouverensis,* which is able to increase As-accumulation of *P. vittata*, grows better in the presence of *Pteris* root exudates compared to other organic acids.

In the rhizosphere of plants grown in As contaminated matrices (soils or waters), the main bacterial genera associated with roots are *Bacillus, Pseudomonas, Lysinibacillus, Acinetobacter, Arthrobacter*. Some of these genera, which are able to resist high As concentrations, are associated with *P. vittata* rhizosphere and are able to increase As uptake by this plant. Indeed, mixed inoculations of bacteria indigenous to a contaminated site can increase As accumulation and biomass of *P. vittata*, thus reducing As concentration in the soil ^21,22^.

Within this framework, the aim of this study was to evaluate, by means of μ-XRF and ICP-OES, As accumulation in plants grown on a naturally As-rich soil in the Viterbo area named Bagnaccio (Lazio region, Italy). Thus, the soil was characterized for its chemical properties, total As concentration, and As bioavailability. The results of this study should reveal the capacity of *Pteris vittata* to phytoextract As when grown on this soil. An additional aim was to isolate and characterize As-resistant bacteria colonising the rhizosphere of *P. vittata* and compare them to those previously associated to *P. vittata* roots grown on polluted soils.

## Results

### Soil characteristics

A summary of Bagnaccio soil properties is shown in Table 1. The soil was characterized by the following components: sandy 46%, clay 20% and loam 34% of granulometric fraction with a sandy clay loam texture (USDA) and a high amount of carbonate reaching values of 59.5%. Soil pH was slightly sub alkaline (pH 7.3) and the exchangeable pH shows a very weak acid reaction with a ∆pH 0.1 (due to the net negative charge of the soil) and a high cation exchangeable capacity.

**Table 1:** Soil quality in Bagnaccio soil: phisical-chemical properties (a) and elemental composition (b).

a		
Parameter	Value	St.deviation
Texture	Sandy clay loam	–
Carbonate (%)	59.5	0.71
pH (H2O)	7.4	0.01
pH (KCl)	7.3	0.01
P available μg/g	0.6	0.08
CEC (meq*100 g)	31.6	0.88
Electrical conductivity µS cm^−1^	3320	35.36
TOC (C%)	1.90	–
Organic Matter (%)	3.1	–
N Total g kg^−1^	1.14	–
C/N	13.47	–
K exchangeable mg kg^−1^	155.4	1.686
Mg exchangeable mg kg^−1^	741.3	3.439
Na exchangeable mg kg^−1^	46.3	0.342
Ca exchangeable mg kg^−1^	3263.1	97.59
Fe exchangeable mg kg^−1^	20.5	0.184

b		
Element	Total element’s quantity mg kg^−1^	St.error
As	750.11	22.0
K	3061.9	130.9
Mg	26,977.8	425.8
Na	1280.6	55.8
Ca	434,227.2	28,432.2
P	1646.1	718.6
Si	3704.5	591.4
Al	33,729.2	2116.9
Fe	28,592.2	2125.0
Mn	12,746.0	776.6
S	358,060.5	19,877.7

Standard deviations or standard errors, as indicated, are reported.

The Total Organic Carbon quantity is good (3.1%), in this volcanic soil that has not been exploited for agricultural purposes or undergone any anthropogenic pollution.

Exchangeable cations show medium values; however, the sodium value is slightly low thus resulting in a high cation exchange capacity. The total average As concentration is 750 mg kg^−1^. Other elements were measured and Ca and Fe values were found to be high, but this is compatible with it being a volcanic and calcareous soil.

**Figure 1:**
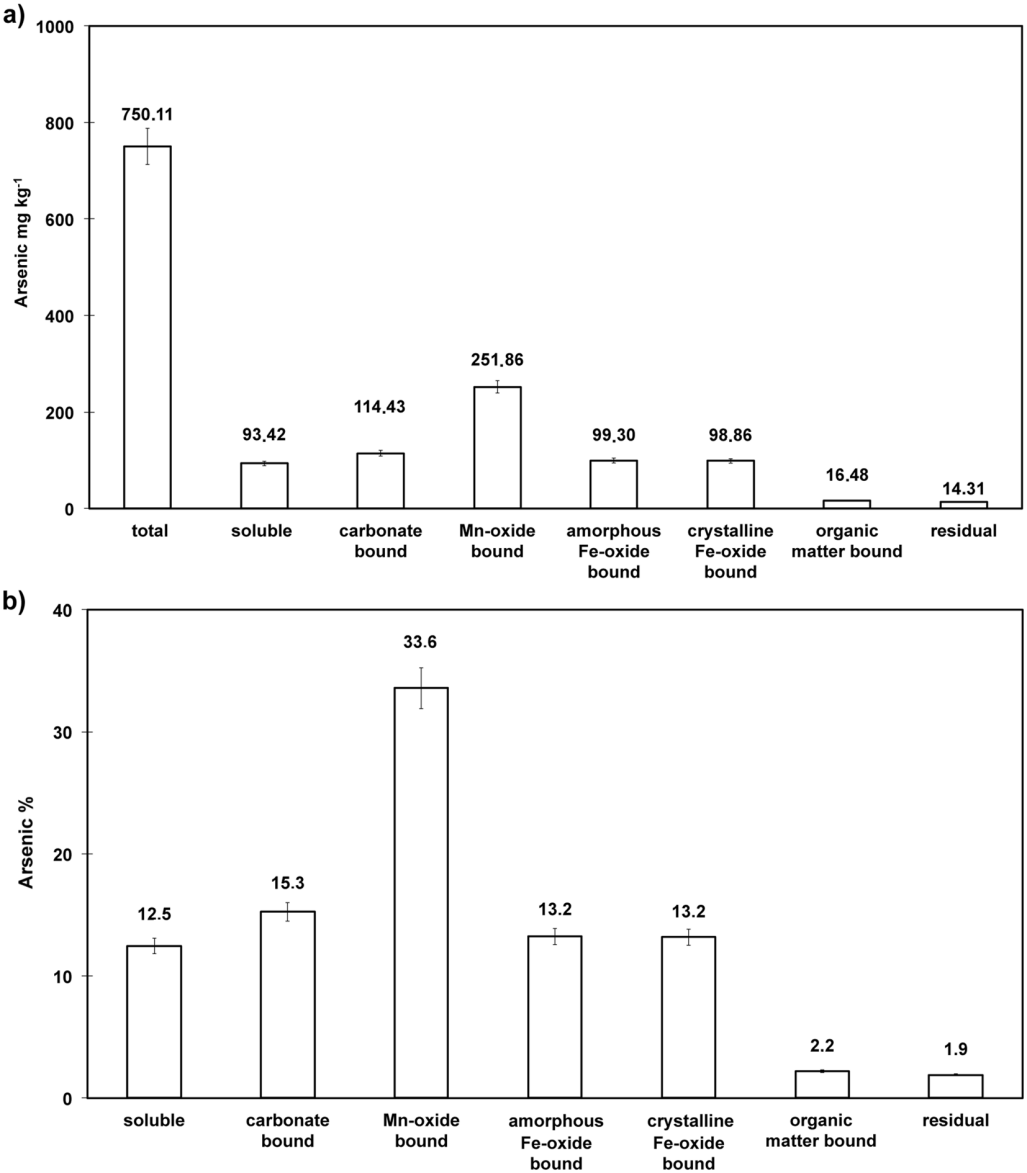
Arsenic amount in the different extracted-fractions released by the SEP of Bagnaccio soil. (**a**) The following pools of soil by SEP 52 were differentiated: As soluble in MgCl2, As bound to carbonate, As bound to Mn-oxide, As bound to amorphous Fe-oxide, As bound to crystalline Fe oxide, As bound to organic matter and sulphide and residual arsenic. All analysis was performed in triplicate. Bioavailable As was counted as the sum of As soluble in MgCl2 and As bound to carbonate; (**b**) results are presented as the percentage of As in each soil fraction after SEP extraction.

To determine the amount of bioavailable As present in the soil, a sequential extraction was performed ^6^, and Fig. 1 shows the distribution of As in the seven soil fractions. Arsenic has a relatively high mobility of 28% resulting from the sum of the first two fractions: soluble and bound to carbonate. Between the seven fractions, the highest total As content was bound with Mn oxides (34%). Arsenic extracted from the fraction associated to amorphous and crystalline iron oxides was low, (13% each). Arsenic percentage extracted from the oxidable fraction (associated to organic matter and sulphur) was very low (2%). Therefore, As is not combined with organic matter or sulphur to a significant extent, and As in residual fractions is very low.

### *P. vittata* plants grown on Bagnaccio soil accumulate high amounts of As

**Figure 2:**
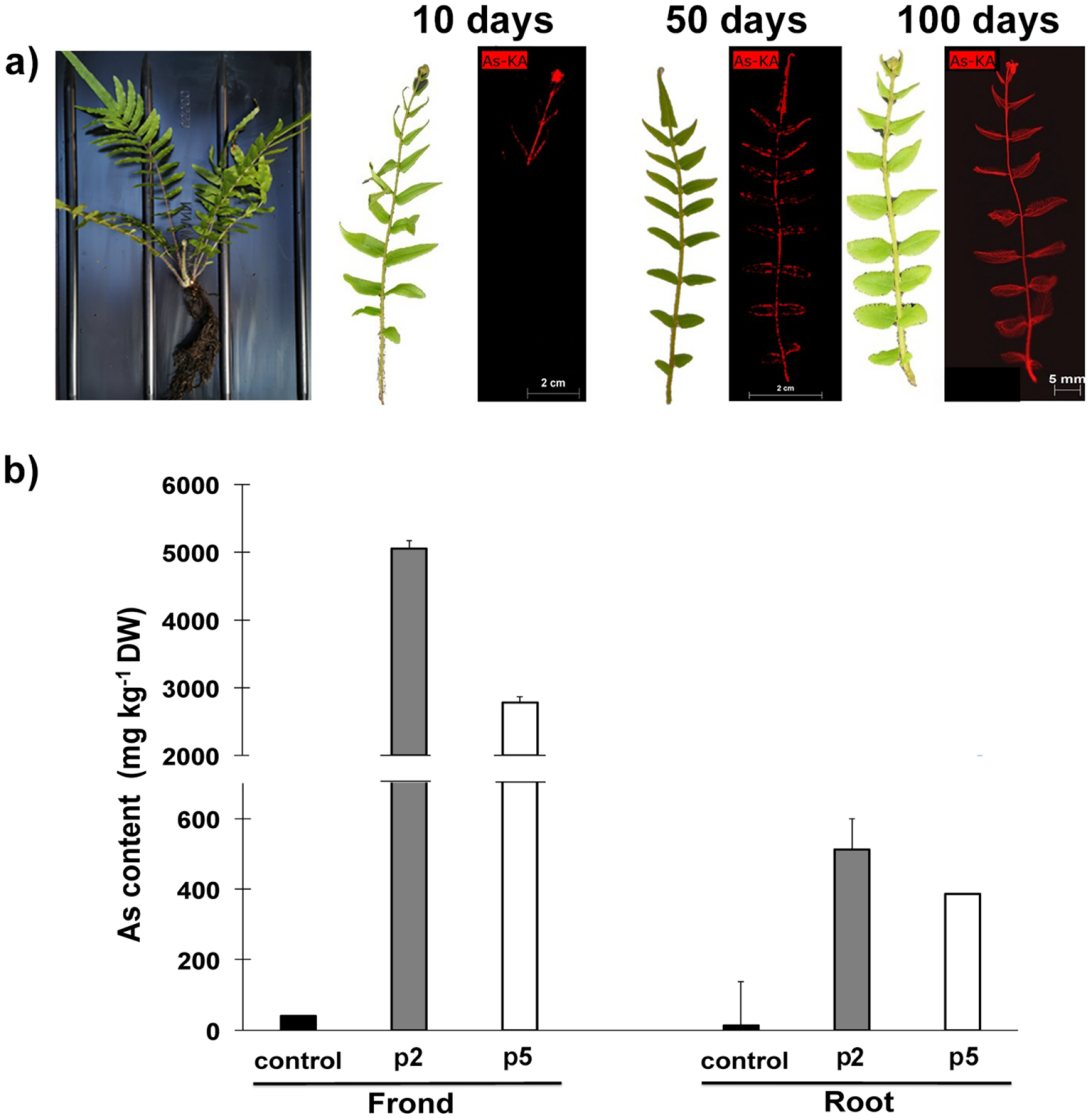
Arsenic distribution and concentration in *P. vittata* plants grown on Bagnaccio soil. (**a**) A 6 month-old Pteris plant (left panel) and µ-XRF elemental maps of fronds from *Pteris vittata* p1, p2 and p5 plants grown for different times on Bagnaccio soil (right panel); (**b**) Arsenic concentration in fronds and roots from *Pteris vittata* p2 and p5 plants grown for 5.5 months on Bagnaccio soil and control plants.

Six-month-old *P. vittata* plants were transferred into Bagnaccio or control non-contaminated soil, and grown under greenhouse conditions. Young fronds were collected from three different plants, named p1, p2 and p5, after 10, 50 and 100 days, respectively and analyzed by µ-XRF technique. As expected, As was not detectable in fronds from plants grown in control soil while it was detectable in the fronds from *P. vittata* plants grown just 10 days on As-contaminated soil (Fig. 2a). After 50 or 100 days, As was detectable in the whole fronds and was preferentially located along the veins and in the apical and marginal regions of the pinna, in agreement with previous reports ^23,24^.

After 5.5 months, bacterial species colonizing *P. vittata* roots were isolated from all three plants, and from plants grown on control soil and subsequently As content in the different organs of p2 and p5 (p1 left alive for further analysis if necessary) plants was analyzed by ICP-OES. As shown in Fig. 2b, As concentration was generally high and was higher in fronds than in roots from both p2 and p5 plants, as expected for this hyperaccumulator.

To assess the capacity of the plant to bioconcentrate As, we calculated the BAF value, that in *P. vittata* is usually greater than 10 ^25^. We found that after 5.5 months of growth in Bagnaccio soil, the BAF_frond_ value was 24.29, while that of BAF_root_ was 2.46.

These results confirm that *P. vittata* is able to accumulate a high amount of As when grown of Bagnaccio soil.

To assess the effects of *P. vittata* growth on the pH of the soil, we compared pH values before planting and after harvesting *P. vittata*. The pH was higher after harvesting (i.e. 7.92 ± 0.02 SE, compared with 7.48 ± 0.03 SE P < 0.01), indicating an increase caused by *P. vittata* growth.

### Taxonomy and phenotypic analysis of bacterial isolates

To identify bacterial species associated to *P. vittata* roots grown on Bagnaccio soil, bacteria were isolated from the soil adhering to *P. vittata* roots. A cultivation-dependent approach was chosen to identify the bacterial species colonizing *P. vittata*. Several strains, which were not detectable, by morphological screening, in *P. vittata* roots grown on uncontaminated soil, were isolated. Sixteen showing distinguishable morphologies were taxonomically typed by using Amplified Ribosomal DNA Restriction Analysis (ARDRA) of the 16S rDNA (Fig. S1 and Fig. S2) that was subsequently sequenced. Phylogenetic analysis indicated that 12 of the 16 isolates can be ascribed to *Bacillus* species*,* and the other four were homologous to *Paenarthrobacter ureafaciens, Beijerinckia fluminensis, Lysinibacillus parviboronicapiens, Acinetobacter schindleri* (Table 2). To assess As tolerance of these species, the 16 bacteria strains were grown at different As concentrations (from 10 to 100 mM AsV). All the isolates, with the exception of PVr_6 and PVr_12 that were sensible to AsV, were tolerant to 10 mM AsV, while strains PVr_2, PVr_5, PVr_9, PVr_15, PVr_16, PVr_17 were resistant to 100 mM AsV (Table 2).

**Table 2:** Characterization of bacterial isolates from *Pteris vittata* roots and their resistance to increasing As (V) concentrations.

Isolate	Gene bank accession nr	As (V)						Homology (%)
		1 mM	3 mM	6 mM	10 mM	75 mM	100 mM	
PVr_1	MT013507	+ + +	+ +	+ +	+ +	nd	nd	*Bacillus halotolerans* (99.75%)
PVr_2	MT013508	+ + +	+ + +	+ + +	+ + +	+ + +	+ +	*Bacillus simplex* (99.34%)
PVr_3	MT013509	+ + +	+ + +	+ + +	+ + +	nd	nd	*Bacillus subtilis* (98.83%)
PVr_5	MT013510	+ + +	+ + +	+ + +	+ + +	+ + +	+ + +	*Paenarthrobacter ureafaciens* (98.16%)
PVr_6	MT013511	+ /‐	nd	nd	nd	nd	nd	*Bacillus amyloliquefaciens* (98.73%)
PVr_7	MT013512	+ + +	+ + +	+ + +	+ +	nd	nd	*Bacillus mojavensis* (99.48%)
PVr_8	MT013513	+ + +	+ + +	+ + +	+ +	nd	nd	*Bacillus mojavensis* (98.92%)
PVr_9	MT013514	+ + +	+ + +	+ + +	+ + +	+ + +	+ + +	*Beijerinckia fluminensis* (100%)
PVr_10	MT013515	+ + +	+ + +	+ + +	+ + +	‐	‐	*Bacillus haynesii* (99.12%)
PVr_11	MT013516	+ + +	+ + +	(+ + +)	(+ + +)	nd	nd	*Lysinibacillus parviboronicapiens* (98%)
PVr_12	MT013517	+ /‐	‐	nd	nd	nd	nd	*Bacillus nealsonii* (98.94%)
PVr_13	MT013518	+ + +	+ + +	+ + +	+ + +	nd	nd	*Bacillus subtilis* (100%)
PVr_14	MT013519	+ + +	+ + +	+ + +	+ + +	nd	nd	*Bacillus subtilis* (99.85%)
PVr_15	MT013520	+ + +	+ + +	+ + +	+ + +	+ +	+ +	*Acinetobacter schindleri* (97.59%)
PVr_16	MT013521	+ + +	+ + +	+ + +	+ + +	+ + +	+ + +	*Acinetobacter schindleri* (98.07%)
PVr_17	MT013522	+ + +	+ + +	+ +	+ +	+	+	*Bacillus halosaccharovorans* (100%)

PVr = *Pteris vittata* root.

Bacterial growth (visual evaluation) after 24 to 48 h in PCA plates at increasing As(V) concentration: ‐absence; + ‐ slight; + moderate; +  + medium; +  +  + high (compared to unpolluted controls) nd = not determined.

### Plant growth promoting activities of the As-resistant bacteria

To assess whether bacterial isolates can promote plant growth, the 16 isolates were assayed for a number of properties relevant to plant growth promoting activities. The qualitative and quantitative test for siderophore production showed that most bacterial strains were able to produce siderophore; in particular PVr_5, PVr_9 strains produced the largest amount of siderophore and were the only strains capable of producing IAA and showing ACC deaminase activity (Table 3).

**Table 3:** Plant growth promoting activities of bacterial isolated from *Pteris vittata* roots.

Isolate	IAA production (mg L^−^^1^)	Siderophore production (psu)	ACC deaminase activity
PVr_1	–	25.67 ± 2.66	–
PVr_2	10.06 ± 0.74	49.55 ± 1.81	–
PVr_3	7.83 ± 0.53	38.78 ± 1.02	–
PVr_5	62.48 ± 6.30	88.64 ± 0.74	+
PVr_6	5.82 ± 2.37	37.69 ± 1.33	–
PVr_7	–	23.31 ± 4.24	–
PVr_8	–	38.83 ± 5.76	–
PVr_9	82.08 ± 1.74	91.90 ± 0.11	+
PVr_10	–	10.22 ± 2.55	–
PVr_11	–	−	–
PVr_12	6.10 ± 0.59	22.39 ± 5.17	–
PVr_13	5.83 ± 1.42	36.61 ± 5.03	–
PVr_14	8.66 ± 3.97	39.07 ± 11.22	–
PVr_15	–	–	–
PVr_16	–	–	–
PVr_17	–	–	

Data are averages of three independent experiments ± S.D. IAA production: (-) < 5 mg L^−1^.

Siderophore production: (−) < 10 psu.

ACC deaminase activity: (−) no bacterial growth on medium containing 1-aminocyclopropane-1-carboxylate as the only N source; (+) bacterial growth on medium containing 1-aminocyclopropane-1-carboxylate as the only N source.

### Various isolates harbour the bacterial gene *arsC*

We also assessed whether the isolates contain the *arsC* gene, responsible for AsVreductase activity. A region of the *arsC* gene was successfully PCR-amplified from the genome of bacterial strains homologous to *Bacillus subtilis* (PVr_3, PVr_13, PVr_14) to *Acinetobacter schindleri* (PVr_15), and to *Paenarhrobacter* genus (PVr_5)*.* No positive PCR products, using primers designed on the *arsC* gene of *Bacillus subtilis,* were found in the other strains showing homology to *Bacillus* genus. This data show that at least five isolates contain the *Bacillus arsC* gene required for the reduction of AsV to AsIII.

## Discussion

In this study we characterized Bagnaccio natural As-rich soil of volcanic origin, we evaluated the As accumulation capacity of *P. vittata* plants grown on this soil under greenhouse conditions, and we isolated and characterized bacteria from *P. vittata* rhizosphere.

Bagnaccio is located near Viterbo in central Italy, where a high concentration of As in soils, not related to anthropogenic contamination, was detected. We found that the Bagnaccio soil shows a composition compatible with its volcanic origin and the non-use as an agricultural soil. Thus, this calcareous soil shows a high amount of total elements. In addition, it has a good quantity of exchangeable elements and organic matter, and this latter property allows plants to grow on this soil.

We found in Bagnaccio soil a high total As content, with an average value of 750 mg kg^−1^ which is not indicative of the real As toxicity towards soil microorganisms and thus the actual soil functionality, As bioavailability and its mobility in the soil ^26,27^. Indeed, we showed by the sequential chemical fractionation analysis that in this soil only 28% of As is bioavailable, i.e. in an easily soluble and leachable form and bound to carbonate. A major portion of As in the soil (34%) is bound to manganese oxides, which are strong oxidants in soils with high sorption capacities. This constitutes a potentially available form depending on the conditions of the surrounding environment. A limited amount of As bound to Fe oxides is also detectable (13%).

In aerobic soils, As is predominantly present in its inorganic form and pentavalent state and is tightly bound to soil particles ^2,28^. In this study, aerobic soil was collected to a depth of 20 cm and used to grow *P. vittata* plants.

It is known that As availability in the rhizosphere is controlled by soil and plant properties ^29,30^. Here, we show that growing *P. vittata* plants for 5.5 months causes a 0.4-unit increase in the rhizosphere pH, in agreement with what was previously observed by Gonzaga et al. ^14^, due to the capacity of *P. vittata* to secrete hydroxyl groups into the soil. This pH increase causes the prevalence of hydrogen arsenate (HAsO_4_^-^) in the soil and increases negative surface charges of soil minerals (such as Fe, Mn, Al oxides), thus possibly inducing a greater mobility of As.

In addition to inorganic ions, *P. vittata* roots secrete a variety of organic compounds, such as phytate and oxalate which can influence the availability of As. In particular, phytate exudates could contribute to the dissolution of Mn oxides, thus increasing As availability in this soil ^20,31^.

We established that *P. vittata* plants, which tolerate very high As concentrations, can grow on Bagnaccio soil and efficiently accumulate As as shown by the high BAF_frond_ value. By means of µ-XRF analysis, we were able to detect As in young fronds as early as after 10 days of growth. Previously early detection of As (before 1 months) have been obtained only in *P. vittata* grown under hydroponic conditions ^32^.

After 5.5 months of growth on Bagnaccio soil, *P. vittata* fronds were able to accumulate 2000–5000 g kg^−1^ of As, as measured by ICP-OES, with a fronds/roots ratio from 10 to 17. It has been previously shown that the fronds/roots ratio, as well as the accumulation capacity, are related to the amount of As in the soil. Interestingly, we observed in plants grown on this natural As-rich soil a fronds/roots ratio, as well as an As content in fronds, similar to those reported for *P. vittata* plants grown for 6 months on a soil supplemented with a high As amount (228 mg kg^−1^)^13^. This roughly corresponds to the amount bioavailable in our soil.

In agreement with the presence of high AsV in Bagnaccio soil, we found that 14 out of the 16 bacterial species isolated from the *P. vittata* rhizosphere are resistant to AsV. Among them, five isolates belonging to different genera (homologous to *Bacillus simplex*, *Paenarthrobacter ureafaciens, Beijerinckia fluminensis* and to two *Acinetobacter schindleri*) are resistant to high concentrations of AsV (> 100 mM AsV).

As determined by genomic PCR analysis, five isolates (homologous to *Bacillus subtilis*, *Acinetobacter schindleri* and *Paenarthrobacter ureafaciens*) contain the *arsC* gene coding for an AsVreductase ^33^, the key enzyme in AsV reduction to AsIII a form that can be extruded by bacterial cells. This process helps bacteria keep As out of the cells when AsV is the dominant species in the medium and enhances As accumulation by plants ^19^.

The majority of our bacterial isolates belong to the *Bacillus* genus, and indeed *Bacilli* have been frequently isolated from *P. vittata* roots grown on As or metal-polluted soils ^34^. Among our isolates, two are homologous to *Bacillus subtilis* and *Bacillus nealsonii* that have been previously associated with As-contaminated soils ^35,36^, while the other two, homologous to *Bacillus simplex* and *Bacillus amyloliquefaciens,* have been associated to soils contaminated by heavy metals such as Cu, Pb, Cd and Cr ^37,38^.

Interestingly most of the *Bacillus* isolates we isolated have not been previously associated with As contamination, thus suggesting that autochthonous bacteria from this soil under study may also have additional roles. Indeed, three strains which showed homology with *Bacillus halosaccharovorans*, *Bacillus halotolerans* and *Bacillus haynesii,* as well as one homologous to *Lysinibacillus* genus, were frequently isolated from saline or hypersaline environments or semi-arid soils and have been shown to grow under stressful conditions for the plant ^39,40^. Halophilic bacilli have an intrinsic high resistance to AsV; thus, their presence in the rhizosphere of plants grown in the Bagnaccio soil, where Na concentration is low, suggests that these bacteria may help plants withstand both high salt and high As concentrations ^41^. Moreover, three isolates showed high homology with *Bacillus mojavensis*, *Bacillus amyloliquefaciens* and *Bacillus halotolerans,* species with a known biocontrol activity: they produce molecules with antibacterial properties, allowing roots to avoid pathogen attack, and they have been found in the rhizosphere of plant living in hostile environments ^42^.

We also found that isolates with homology to *Paenarthrobacter ureafaciens* and *Beijerinckia fluminensis* produced high levels of IAA and of ACC deaminase, an enzyme responsible for ethylene production. This is in agreement with previous evidence suggesting that bacterial IAA/ethylene facilitates the adaptation of host plants to metal or As-contaminated sites ^43^. We also showed that these bacteria, which have never been previously isolated from *P. vittata* roots, produce siderophores, a property that has not been previously described for bacteria homologous to *Paenarthrobacter urea* and *Beijerinckia fluminensis* ^44^. The production of siderophore can enhance As tolerance by improving Fe nutrition, or can also increase As uptake as they can solubilize Fe-As minerals ^45,46^.

## Conclusions

In this work, we show that *P. vittata* efficiently phytoextracts As from a volcanic soil with a high concentration of total and bioavailable As. In agreement, we were able by means of µ-XRF, to detect As very early in *P. vittata* fronds. Our data open the possibility to a phytoextraction strategy in this heavily, naturally As contaminated area.

Our data also show that the rhizosphere of *P. vittata* is colonized by several As-resistant bacteria, most of them belonging to the genus Bacillus, and not previously associated to As in polluted soils. Future work will be necessary to dissect the complexity of the roles of the bacterial strains recruited by *P. vittata* roots, which could mitigate As toxicity, promote *P. vittata* growth and/or As uptake ability.

## Material and methods

### Site description, sample collection

**Figure 3:**
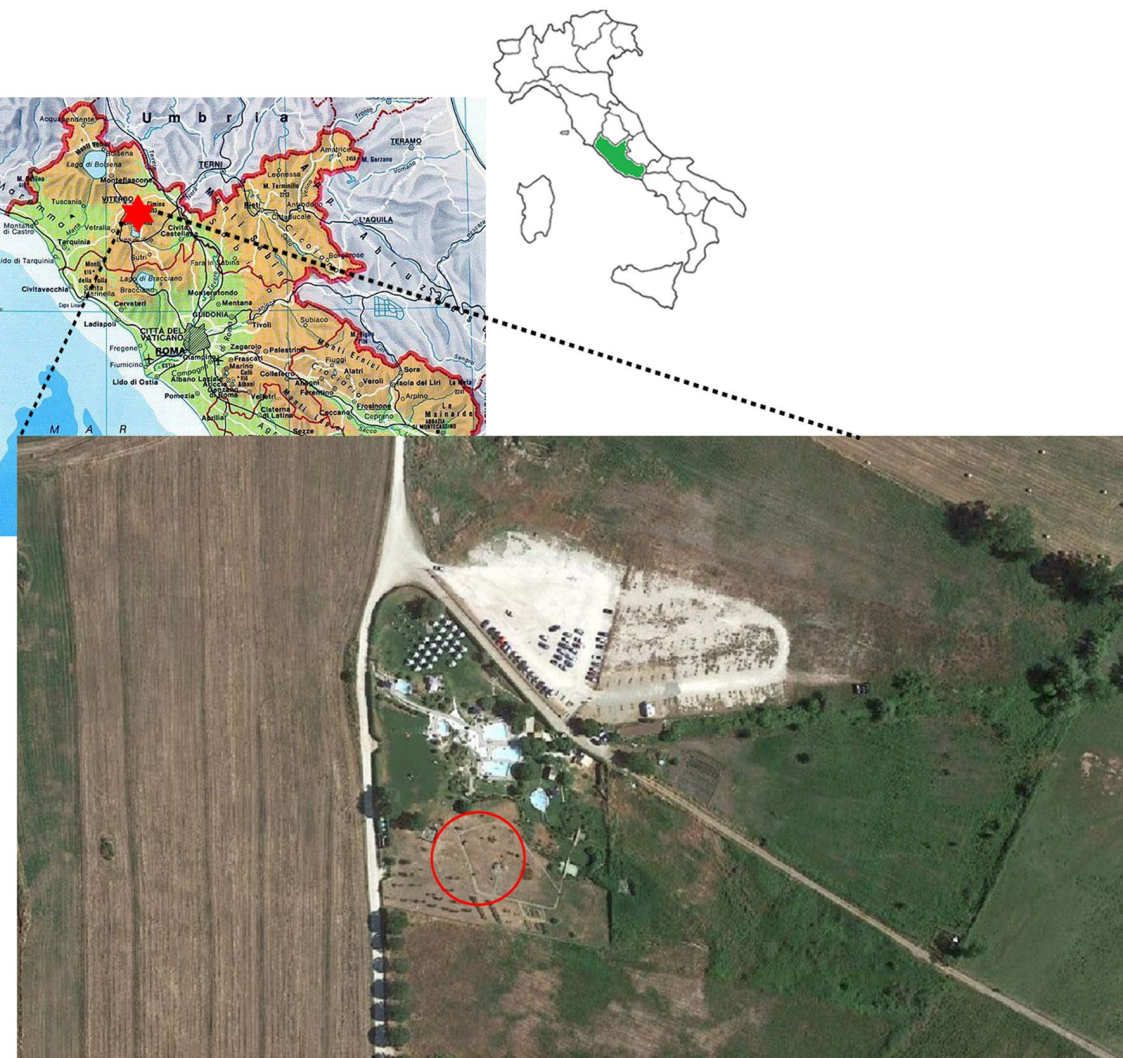
Location of the Bagnaccio soil used in this study. A detailed map of the Lazio region (in green in the map of Italy) is shown and the Bagnaccio area is indicated by a star. The circle on the enlarged image indicates the specific soil under study.

Viterbo is a naturally As-rich volcanic area. Arsenic abundance and mobilization in this zone are a result of hydrothermal processes that cause the up-flow of thermal waters, and As concentrations range between 180 to 370 µg L^−1^. Soil samples were collected from Bagnaccio, an area situated in the western side of Viterbo (Lazio, Italy) (42°27′30.4″ N 12°03′55.9″ E) at a depth of 0–20 cm, during Spring 2019 (Fig. 3). A 10 × 10 m homogeneous area was chosen, three soil cores were taken at a maximum distance of 1 m from each other. Soil samples were partly used for physical–chemical characterization and partly for plant growth experiments.

### Pre-treatment of soil samples and chemical characterization

Soil samples were sieved (< 2 mm) and oven dried at a room temperature.

Total organic carbon (TOC) was determined by dry combustion ^47^ using an elemental analyzer (Shimadzu TOC VCSH analyzer). Total carbonate content was measured by the Calcimetric method. Active CaCO_3_ was determined with 0.1 M NH_4_-oxalate, as described by Drouineau ^48^.

A solution of deionized (active) or 1 N KCl (exchangeable) water, respectively, in a ratio of 1: 2.5 (w / v), was used to measure active and exchangeable acidity in the soil. The pH was measured in the supernatant (pH 211, HANNA instruments, Woonsocket, Rhode Island (USA). Electrical conductivity was measured using the portable device HI9033 (Conductivity meter: HANNA instruments, Woonsocket, Rhode Island (USA).

Cation exchange capacity (CEC) was determined after extraction with 10 percent BaCl_2_ solution pH 8.1 ^49^. Results were expressed as cmol^+^ kg^−1^ of soil. Exchangeable cations (K, Na, Mg, Ca and Fe) were quantified by extraction with 1 M NH_4_OAc at pH 7 and measured by ICP-OES.

All analyzes performed in this study were recorded on the basis of their dry mass. To determine the humidity, the samples were dried to constant mass in an oven at 105 ± 5° C. The difference in mass before and after the drying process is used to determine the dry matter and moisture content.

### Chemical analysis in plant and soil and determination of bioavailable As

The total As amount in plants was determined after mineralization of the samples with concentrated hydrochloric acid (36% HCl), nitric acid (69% HNO_3_) and hydrogen peroxide (30% H_2_O_2_) (Merck, Darmstadt, Germany) with a microwave assisted digestion (Mars plus CEM, Italy) ^50^.

The total concentration of As, K, Mg, Na, Ca, P, Si, Al, Fe, Mn, S, were analyzed in all the collected soil samples. Analyses were performed in triplicate ^8^. As_2_O_3_ standard was purchased from CaPurAn (CPA chem, Bulgaria). The purity of the plasma torch argon was greater than 99.99%.

The accuracies of the measurements were assessed using trace metals loamy sand 3 standard reference materials (CRM034–Fluka).

Total element concentrations were measured by ICP-OES, Optima 8000DV, Perkin Elmer) equipped with a Scott nebulizer was used.

To determine the amount of bioavailable As, a sequential extraction procedure (SPE) was performed. Arsenic was determined in seven diverse fractions of the soil samples ^51^.

### Plant experiment

The propagation and growth of ferns was performed in the greenhouse under controlled conditions ^52^. We illuminated with a 150 µmol s^−1^ m^−2^ white light lamp set to a photoperiod of 16 h/8 h light/dark. Temperature was set at 28 °C and humidity at 70%. Spores were resuspended in water and sown in pots with soil and expanded clay and covered with a thin plastic film. Humidity was maintained constant by daily MilliQ water spraying. After 4 weeks, gametophytes were visible. After 8 weeks, the young sporophytes reached about 2 cm in height and were separated in different pots. 6-month-old ferns were then transplanted into (20 cm-diameter 14 cm-high) plastic pots containing As contaminated soil and young fronds, collected after different times were analyzed by µ-XRF.

The As bioaccumulation factor (BAF) was calculated as the ratio between its concentration in fronds (BAF_frond_) or roots (BAF_root_), and the corresponding bioavailable As concentration in soil ^2^. The use of the bioavailable As concentration was preferred to assess the real risk associated with the presence of As in soils and its uptake by plants.

### μ-XRF

A μ-XRF benchtop spectrometer (M4 Tornado, Bruker), equipped with a Rh X-ray tube with polycapillary optics and XFlash detector providing an energy resolution of better than 145 eV, was utilized to perform the analysis. The sample chamber operates in void conditions (i.e. 25 mbar). Samples preparation was performed following the strategies described in Capobianco et al. ^11^.

Constant exciting energies of 50 kV and 500 μA were applied during the analysis.

The set-up mapping acquisition parameters comprised a pixel size of 60 μm and an acquisition time, for each pixel, of 20 ms.

### Isolation and identification of bacteria from *P. vittata* rhizosphere

*P. vittata* plants were extracted from the soil and roots were scrolled to remove the non-adherent soil from them. Roots were placed in 0.3% sodium pyrophosphate under stirring conditions for about 1 h. Four-fold dilutions were then spread onto plates containing plate count agar (PCA) medium (Sigma Aldrich, St. Louis, MO) and then incubated for 4 days at 28 °C. DNA was extracted from bacterial colonies isolated by diluting each colony in 40 μL of sterile water and exposing it to 3 freezing cycles in liquid nitrogen and thawing at 90 °C as previously described ^53^. Two mL of the released DNA from each colony was amplified by PCR in an Applied Biosystems thermocycler (Foster City, CA, USA) ^54^. 16S rDNA amplified fragments were digested with the restriction endonucleases *Hae* III or *Mps* I overnight at 37 °C. Isolates corresponding to different amplified ribosomal DNA restriction analysis (ARDRA) patterns were selected. The 16S rDNA PCR products were purified using the “Nucleospin Gel and PCR Clean-up” kit (QIAGEN, Hilden, Germany) and subsequently sequenced (http://www.biofabresearch.it/sequenziamento/sequenziamento-gen.html). Partial 16S rDNA sequences were matched against nucleotide sequences with the BLAST tool in the NCBI website. The nucleotide sequences (PVr_1 to PVr_17 colonies) have been deposited in the GenBank database under accession numbers MT013507 through MT013522.

### Plant growth promoting activities of isolates

IAA and 1-aminocyclopropane-1carboxylic (ACC) deaminase production were tested according to Sheng et al. ^55^. Siderophore production was measured according to the chrome azurol S (CAS) analytical method ^56^ on agar plates using a modified CAS (O-CAS) assay described by Pérez-Miranda et al. ^57^.

To quantify siderophore activity, bacterial cultures grown in SMS medium (sucrose minimal salts) for 4 days were centrifuged to remove bacterial cells. 500 μL of supernatant was added to the same volume of CAS solution and was incubated for 20 min at RT. The CAS assay solution was prepared according to Jeong et al. ^58^. The absorbance at 630 nm was determined to quantify the siderophore production by each strain, and the result was expressed as percent siderophore unit ^59^. Two replicates per bacterial colony were analyzed.

### Bacterial *arsC* gene amplification

DNA of bacterial colonies was extracted and used in reactions performed with the ‘GoTaq G2 Colorless Master Mix’ (Promega, Madison, WI USA). The primers used for *arsC* gene amplification were designed on sequences found in NCBI for *Acinetobacter schindleri* (NZ_CP015615.1) 5′-TCCCGAATGTGGAACCTCTC-3′ (forward) and 5′-AAT CGC TTC ACG TAC CGA CA-3′ (reverse), *Bacillus subtilis* (NC_000964.3) 5′- TGC TGA TTT AGT TGT TAC GC -3′ (forward) and 5′- TTC CTT CAA CCT ATT CCC TA-3′ (reverse) and genus *Paenarthrobacter* (NC_008713.1) 5′- AAC GCT ACG TCT TCG AGT CC-3′ (forward) and 5′-GAG TTC TGA TGC GGG TAG GG-3′ (reverse).

## Supplementary Information

Supplementary Information
