# Supplementary material for: Phytoextraction efficiency of Pteris vittata grown on a naturally As-rich soil and characterization of As-resistant rhizosphere bacteria
Source: Sci Rep. 2021 Mar 24;11:6794. doi: 10.1038/s41598-021-86076-7 (PMC7990962; doi:10.1038/s41598-021-86076-7)
Supplement: Supplementary file 1 — Supplementary Information [file 41598_2021_86076_MOESM1_ESM.pdf]

Phytoextraction efficiency of *Pteris vittata* grown on a naturally As-rich soil and characterization of As-resistant rhizosphere bacteria

Antenozio ML[a,b1], Giannelli G[c1], Marabottini R[d], Brunetti P[a], Allevato E[f], Marzi D[a], Capobianco G[e], Bonifazi G[e], Serranti S[e], Visioli G[c], Stazi SR[f*] and Cardarelli M[a*]

[a]IBPM-CNR, Dip. Biologia e Biotecnologie, Sapienza Università di Roma P.le A. Moro 5, 00185 Rome, Italy
[b]Dip. Biologia e Biotecnologie, Sapienza Università di Roma, 00185 Rome, Italy
[c]Department of Chemistry, Life Sciences and Environmental Sustainability, University of Parma, Parco Area delle Scienze 11/A, 43124 Parma, Italy
[d]Department for Innovation in Biological, Agri-Food and Forestry Systems (DIBAF), University of Viterbo, Via San Camillo de Lellis snc, 01100 Viterbo, Italy
[e]Dip. Ingegneria Chimica Materiali Ambiente, Sapienza Università di Roma, 00184 Rome, Italy
[f]Department of Chemical, Pharmaceutical and Agricultural Science (DOCPAS), University of Ferrara I-44121 Ferrara, Italy.

[1] These authors equally contributed to the work and are considered co-firstauthors.

* corresponding authors:
maura.cardarelli@uniroma1.it 0000-0002-8190-8939;
silviarita.stazi@unife.it 0000-0001-6827-3062.

Figure S1

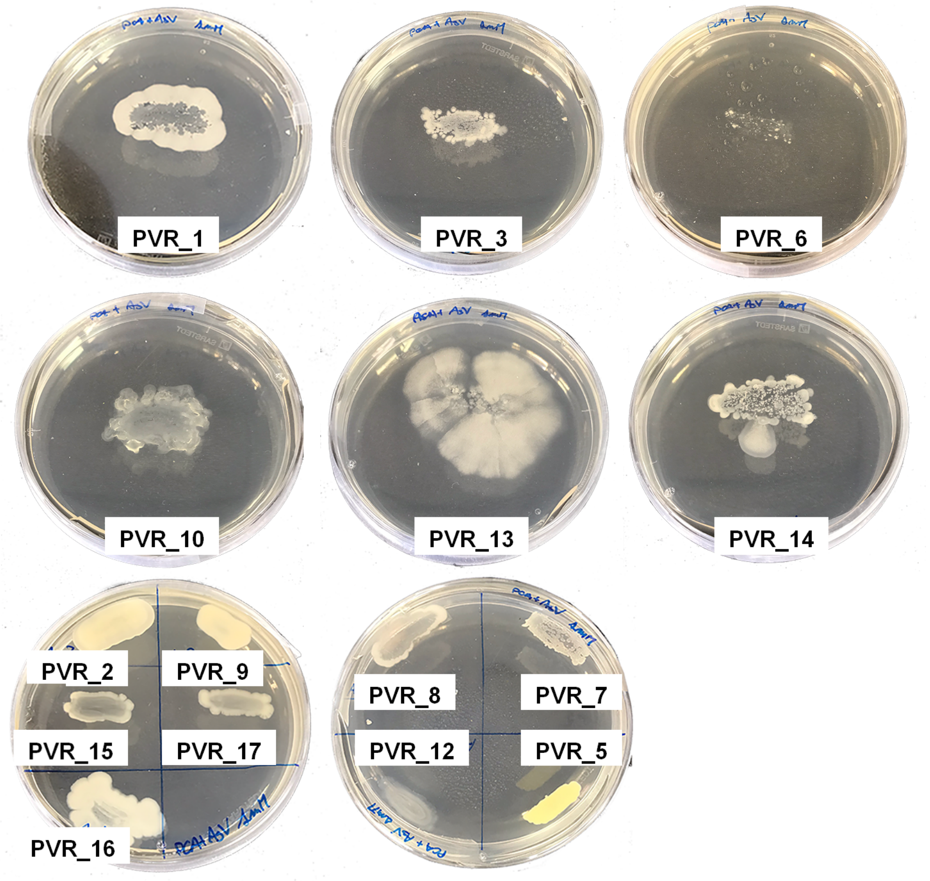

Fig. S1 Sixteen bacterial strains isolated from *Pteris vittata* rhizosphere and resistant to 1mM AsV.

Figure S2

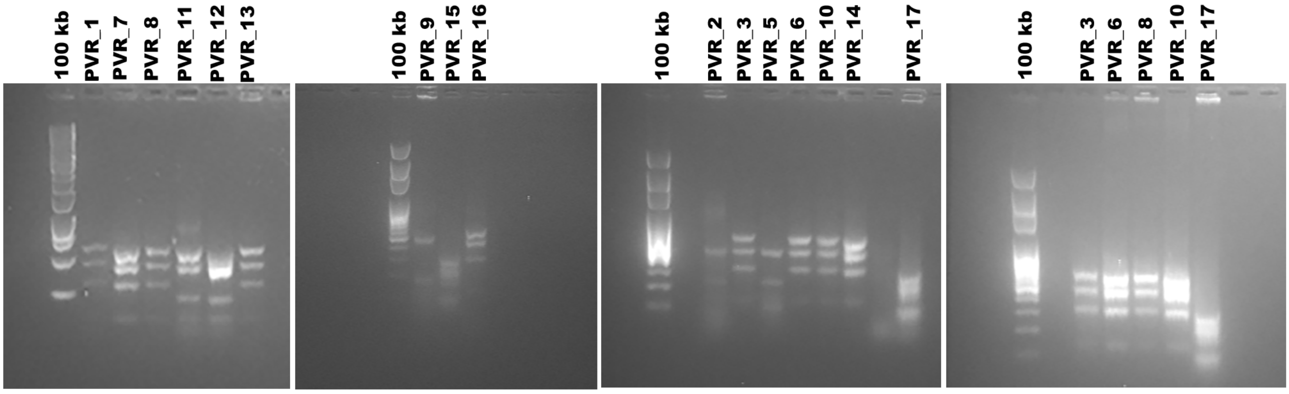

Fig. S2 Comparison of amplified ribosomal DNA restriction analysis (ARDRA) profiles based on the restriction fragments of 16s rDNA by *Hae III* of the 16 bacterial strains isolated by *Pteris vittata* rhizosphere and resistant to 1mM AsV.
